# MicroRNA Exocytosis by Vesicle Fusion in Neuroendocrine Cells

**DOI:** 10.3389/fendo.2017.00355

**Published:** 2017-12-22

**Authors:** Yongsoo Park

**Affiliations:** ^1^Department of Molecular Biology and Genetics, Koç University, Istanbul, Turkey

**Keywords:** microRNA, fusion, chromaffin cells, large dense-core vesicles, neuroendocrine cells, SNARE

## Abstract

MicroRNAs (miRNAs) are short non-coding RNAs that posttranscriptionally regulate gene expression inside the cell. Extracellular circulating miRNAs are also observed outside the cell, but their origin is poorly understood. Recently, miRNA has been shown to be exocytosed by vesicle fusion; this observation demonstrates that vesicle-free miRNAs are secreted from neuroendocrine cells, in a manner similar to hormone secretion. miRNAs are stored in large dense-core vesicles together with catecholamines, then released by vesicle fusion in response to stimulation; in this way, vesicle-free miRNA may regulate cell-to-cell communication including the regulation of gene expression and cellular signaling. Therefore, miRNA has been suggested to function as a hormone; i.e., a ribomone (ribonucleotide + hormone). This review focuses on the mechanisms by which vesicle-free miRNAs are secreted from neuroendocrine cells and will discuss potential functions of vesicle-free miRNAs and how vesicle-free miRNAs regulate cell-to-cell communication.

## Introduction

MicroRNAs (miRNAs) are a class of small non-coding RNAs (ncRNAs) that are ~22 nucleotides in length; they downregulate translation of target mRNA (1, 2). ncRNAs are transcribed from the genome, but not translated to protein; ~98% of RNA transcripts in humans are non-coding (3). Although miRNAs constitute <1% of ncRNAs in mammalian cells (4); tRNA and rRNA are dominant ncRNAs, miRNAs have critical functions in gene expression.

MicroRNAs inhibit expression of >60% of human protein-coding genes, mostly by binding to the 3′- untranslated region (3′UTR) of the target mRNAs (5) and, therefore, miRNAs affect gene expression networks of a variety of biological processes including development, apoptosis, proliferation, and metabolism (1, 2). miRNAs are transcribed within cells, but are also found outside cells, called extracellular miRNAs. Extracellular miRNAs were observed in cell culture system (6), in blood plasma and serum (7–10), and in other biological fluids (11) including cerebrospinal fluid (12), saliva (13), breast milk, urine, and tears (14). The existence of extracellular miRNAs suggests that they participate in cell-to-cell communication. Extracellular miRNAs are highly stable in freeze-thaw cycles, extreme pH, and can withstand storage for up to 4 days at room temperature (9, 10, 15).

Extracellular miRNAs can be non-invasive biomarkers for many different types of diseases (16–19), although the specificity and sensitivity of miRNA biomarkers are still under debate (20), for three reasons: (1) tumor-derived extracellular miRNAs can also be released by normal cells; (2) existing protocols for collecting extracellular miRNAs are not sufficiently reproducible; and (3) the level of tumor-derived extracellular miRNAs might vary with the age of the patient and the status of disease, so their value as non-invasive biomarkers are reduced.

Exosomes, microvesicles, and apoptotic bodies are considered as carriers of extracellular miRNAs (21). More than 90% of extracellular miRNAs are vesicle-free, but form a complex with proteins such as Agonaute2 (AGO2) (22, 23). Although extracellular miRNAs are believed to contribute to cell-to-cell communication, the mechanisms by which miRNAs are released are still not understood. Extracellular miRNAs have been considered as byproducts or artifacts caused by cell lysis and cell death. Recently, miRNA exocytosis by vesicle fusion in response to stimulation was observed in chromaffin cells, which are neuroendocrine cells in the sympathetic nervous system (24). The objective of this review is to discuss how miRNAs are released by active exocytosis and to examine the physiological functions of vesicle-free miRNAs in neuroendocrine cells.

## Carrier of Extracellular miRNAs

The biogenesis of miRNAs has been extensively reviewed elsewhere (25–27). RNA polymerase II mainly transcribes microRNA genes as primary miRNA transcripts (pri-miRNAs) that contain 5′cap and 3′poly(A) tails (28). Drosha, RNase III, and DGCR8, the RNA-binding protein, further process pri-miRNAs into stem-loop structured precursor miRNAs (pre-miRNAs) of ~70 nt (29). After pre-miRNAs are transported to the cytoplasm, RNase III Dicer and TRBP (transactivation-response RNA-binding protein) cleave them into double-stranded miRNA duplexes of ~22 nt (30, 31). Finally, argonaute (AGO) proteins associate with mature miRNAs in the RNA-induced silencing complex (RISC) (32–34) and mature miRNAs bind to the complementary sequence usually located within the 3′-UTR of target mRNAs (35). AGO protein family (AGO1, AGO2, AGO3, AGO4) associating with miRNA mediate mRNA decay and inhibition of mRNA translation, whereas only AGO2 cleaves target mRNAs (32, 36).

Exosomes, microvesicles, and apoptotic bodies deliver extracellular miRNAs to target cells (21). Exosome-incorporated extracellular miRNAs were first observed in 2007 (6). Exosomes contain miRNAs and mediate the transfer of miRNAs between cells (6). Release of exosomal miRNA is dependent on ceramide, which is regulated by neutral sphingomyelinase 2 (nSMase2), but independent of the endosomal sorting complex required for transport (ESCRT) (37, 38). Rab27a and Rab27b control exosome secretion by regulating docking of multivesicular bodies (MVBs) at the plasma membrane (39).

Exosomes are small vesicles (40–100 nm in diameter) and are thought to be the carriers of signaling macromolecules and RNAs for cell-to-cell communication, but the true function of exosomes remain poorly understood (40–42). MVBs store exosomes and release exosomes by fusion with the plasma membrane (43). However, the number of copies of miRNAs per exosome is very low; i.e., <1 (44), and exosomes can be very heterogeneous in molecular composition depending on the purification methods (45). Furthermore, further research is required to determine the mechanism by which exosomal miRNAs (44, 46, 47) affect gene silencing in the target cell, despite their low concentrations [see Table 1 for comparison with large dense-core vesicles (LDCVs)].

**Table 1:** Comparison of large dense-core vesicle (LDCV) and exosome^a^.

	LDCV	Exosome^a^
Size (diameter)	100–300 nm	40–100 nm
Biogenesis/formation	Golgi complex	Multivesicular bodies, endosome
Agonaute2 and RNA-induced silencing complex	No (24)	Yes (48)
Copy number of miRNA	~500 (miR-375) (24)	<1
Size distribution of RNA	Peak at ~22 nt (24)	Broad distribution, 25–4,000 nt (49)
Dominant RNA	~60% miRNA (24)	mRNA, miRNA is minor (<1–30% (49–52))
Contents	Catecholamines, hormones, peptide, ATP, miRNA	Proteins, DNA, RNA, lipid (42, 53–55)
miRNA release mechanism	Neuronal SNARE (VAMP-2, syntaxin-1A, SNAP-25A) (56)	Ceramide-dependent, ESCRT-independent (37, 38)

*^a^Exosome composition is so heterogeneous that the RNA profile and content of exosomes varies depending on cell types, references; i.e., serum, plasma, or cell culture medium, developmental stages, etc*.

Microvesicles from the plasma contain miRNAs and transfer extracellular miRNAs (57). Microvesicles are generated by outward budding of the plasma membrane and are larger (50–2,000 nm) than exosomes (58). The sizes of some microvesicles and exosomes are similar and the molecular compositions of microvesicles and exosomes largely overlap (59), so, the classes are difficult to distinguish; thus, they can be collectively called extracellular vesicles (EVs).

Apoptotic bodies with diameters 1–4 μm contain extracellular miRNAs (60), but apoptotic bodies form only after programmed cell death, and miRNAs in apoptotic bodies seem to be byproducts released by cell lysis. Researchers still debate whether extracellular miRNAs are the specific cargo of EVs and apoptotic bodies, or whether miRNAs are just byproducts of the biogenesis of EVs and apoptotic bodies.

## Extracellular Vesicle-Free miRNAs

Extracellular miRNAs fall into vesicle-incorporated and vesicle-free groups. More surprisingly, 90–99% of extracellular miRNAs are vesicle-free, and are components of miRNA–protein complexes; this result suggests that exosomes are not the main miRNA carriers (22, 23). Extracellular vesicle-free miRNAs being exported by the protein complexes that protect miRNAs from degradation, e.g., nucleophosmin 1 (NPM1), in the cell culture system were first reported in 2010 (61). miR-16 and miR-92a are not contained in EVs, but associate with AGO2 that protect extracellular miRNAs from RNases (22). miR-16, miR-21, and miR-24 are EV-free extracellular miRNAs that form complexes with AGO2 (23). In addition to AGO2, apolipoprotein A-I (apoA-I), the main component of high-density lipoprotein (HDL), associates with extracellular miRNAs in plasma and transfers miRNAs to target cells; the transfer is mediated by a scavenger receptor class B, type I (SR-BI) HDL receptor in the plasma membrane (62, 63), and thereby contributes to intercellular communication.

Despite the interests of extracellular vesicle-free miRNAs, the origin of vesicle-free miRNAs and the mechanisms of their release are unclear. Vesicle-free miRNAs might be byproducts of cell death (23); death of neurons and glial cells in neurodegenerative diseases lead to an increase in extracellular vesicle-free miRNAs, and this increase can be exploited as a biomarker (64). In contrast, certain vesicle-free miRNA species are selectively released from various types of cells (61); this result suggests the existence of a specific pathway that is independent of EVs for release of miRNAs. However, the mechanisms by which vesicle-free miRNA is released remain unknown.

## miRNA Exocytosis by Vesicle Fusion

**Figure 1:**
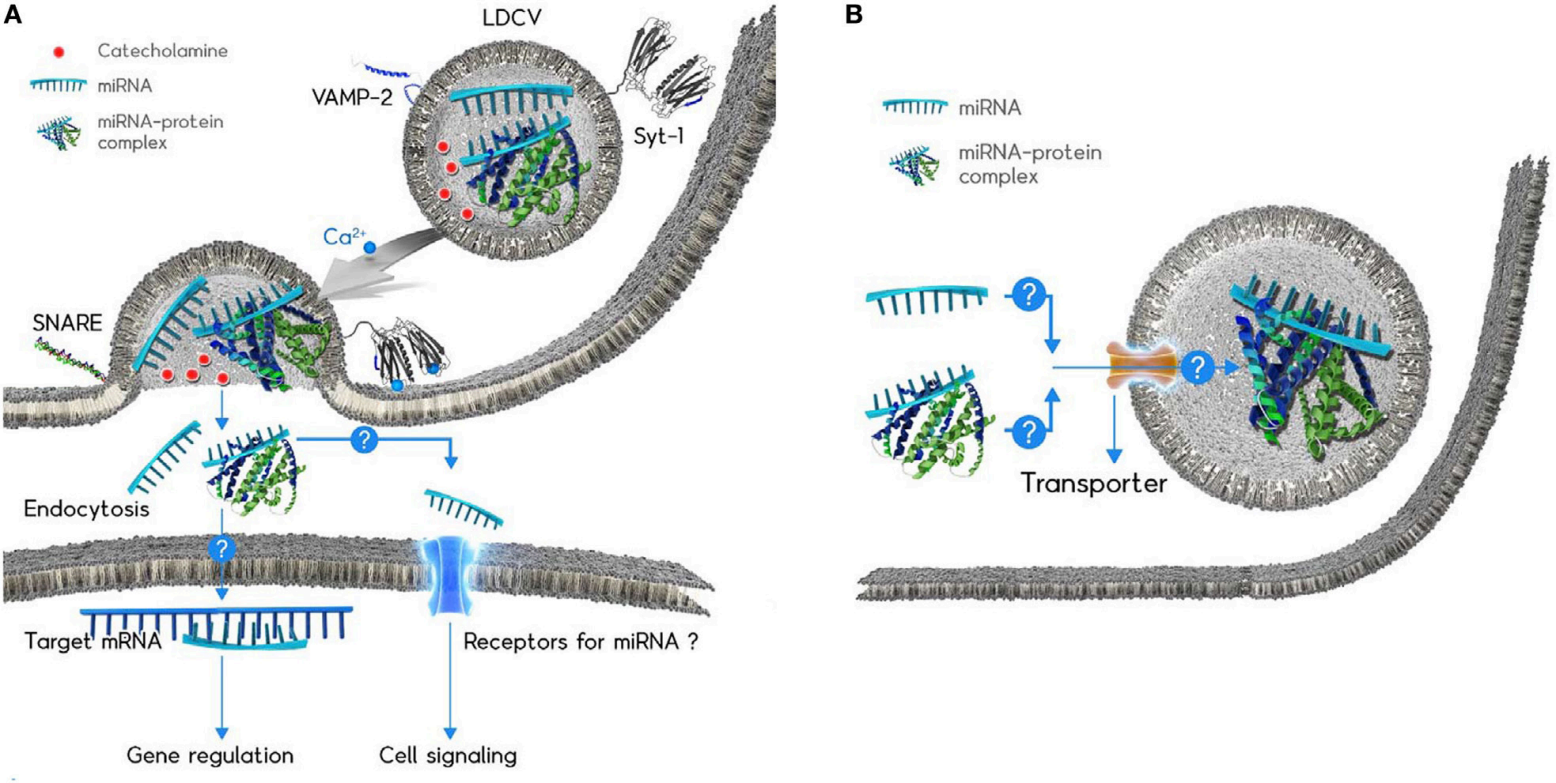
Schematic diagram of the microRNAs (miRNA) exocytosis mechanisms **(A)** and the working hypothesis of the miRNA loading into large dense-core vesicles (LDCVs) **(B)**. **(A)** Catecholamines (red ball) are typical neurotransmitters stored in LDCVs. LDCVs also contain a variety of miRNAs including miR-375. The assembly of neuronal SNAREs including VAMP-2, SNAP-25A, and syntaxin-1A mediates miRNA exocytosis from chromaffin cells, neuroendocrine cells. Synaptotagmin-1 (Syt-1) is considered as a Ca^2+^ (green ball) sensor to trigger miRNA exocytosis. The membrane insertion of Ca^2+^-bound Syt-1 results in the fusion pore formation. Ribomone hypothesis: miRNAs stored in vesicles together with classical neurotransmitters are released by vesicle fusion, thereby contributing to cell-to-cell communication (24). Two hypothetical functions of released extracellular miRNAs; (i) miRNAs might be taken up by endocytosis into target cells where miRNAs regulate gene expression. (ii) miRNAs might be able to stimulate receptors or ion channels as ligands, thereby leading to cellular signalling. Adapted from Gümürdü et al. (24). **(B)** The mechanisms by which miRNA or miRNA–protein complex can be loaded into LDCVs remain elusive. Structure of miRNA-binding protein is artificial for the simplicity.

Neuroendocrine cells can release vesicle-free miRNAs by active exocytosis in response to neuronal stimuli (24). Chromaffin cells are neuroendocrine cells that release hormones and catecholamines (e.g., dopamine, adrenaline, noradrenaline) into the blood when the sympathetic nervous system is activated (65). LDCVs of chromaffin cells are specialized organelles that store catecholamines and hormones (65). miRNAs are stored in LDCVs of chromaffin cells together with catecholamines and hormones (see Table 1 for comparison with exosome); miR-375 is the most abundant miRNA (~30% of total miRNAs in LDCV) (24). miRNAs including miR-375 are released by LDCV fusion in a manner that is dependent on the presence of soluble *N*-ethylmaleimide-sensitive factor attachment protein receptor (SNARE). SNARE proteins are considered to constitute the fusion machinery that draws two opposing membranes close together (66). Neuronal SNAREs including VAMP-2, SNAP-25A, and syntaxin-1A mediate miRNA exocytosis in neuroendocrine cells (Figure 1A) (24), whereas VAMP-3 and SNAP-23 mediate secretion of vesicle-free miRNA in vascular endothelial cells (67). miRNA exocytosis is completely inhibited when neuronal SNAREs are absent in the *in vitro* reconstitution system, suggesting that neuronal SNAREs mediate the release of miRNAs in chromaffin cells (24).

Ca^2+^ is a triggering factor of vesicle fusion and synaptotagmin-1 (Syt-1) is a Ca^2+^ sensor for fast exocytosis in neurons (68) and neuroendocrine cells including chromaffin cells (56). The membrane insertion of Syt-1 into the plasma membrane triggers Ca^2+^-dependent vesicle fusion (69). miR-375 exocytosis is accelerated by the Ca^2+^ influx that provokes LDCV fusion in PC-12 cells, the cell line of chromaffin cells as well as the *in vitro* reconstitution system (24); this observation is evidence that miRNA exocytosis is coupled to neuronal stimuli, and that Syt-1 is a Ca^2+^ sensor for miRNA exocytosis in neuroendocrine cells (Figure 1A).

Large dense-core vesicles are enriched with miRNAs that account for ~60% of total RNAs stored in LDCVs; the copy number of miR-375 stored in a single LDCV is ~500 (24), which is extremely high compared to the copy number (<1) in exosomes (44, 46) (see Table 1). miR-375 is preferentially stored in LDCVs in chromaffin cells, but not in synaptic vesicles in neurons (24); this segregation suggests that miRNA exocytosis by LDCV fusion is specific. Thus, a new term: ribomone (ribonucleotide + hormone) has been proposed; i.e., miRNA can function as a hormone, which is stored in vesicles and released by vesicle fusion together with neurotransmitters in response to stimulation, and in this way, contributes to cell-to-cell communication (24).

Vesicle-free miRNAs are highly stable. One possibility is they are stabilized by RNA-binding proteins outside the cells, e.g., by AGO2 (22, 23), apoA-I (62), and NPM1 (61). The mechanism of this stabilization in LDCVs after exocytosis remains unknown, but two hypotheses can be proposed. LDCVs contain apoA-I, but neither AGO2 nor NPM1 (24), thereby, it remains to be tested that apoA-I binds and stabilizes miRNAs. Another possibility is that secreted miRNAs bind to AGO2 that exists outside the cells and AGO2 might stabilize secreted miRNAs. We also cannot exclude the possibility that other RNA-binding proteins might be involved in miRNA stability.

miR-375 is specifically expressed in endocrine and neuroendocrine cells, including pancreatic islets beta-cells, pituitary gland, and adrenal medulla chromaffin cells (70, 71); miR-375 is specifically located in the intermediate lobe of pituitary (72). Organs and cells expressing miR-375 are linked in hormone secretion. miR-375 inhibits catecholamine biogenesis by reducing the expression of tyrosine hydroxylase and dopamine-beta-hydroxylase in chromaffin cells (73). miR-375 is one of the first miRNAs that was identified in the pancreas; miR-375 regulates development of pancreatic islets (74) and normal pancreatic cell mass (71). miR-375 also reduces insulin secretion by suppressing expression of myotrophin (70) and phosphoinositide-dependent protein kinase-1 (PDK1) (75). In the pituitary gland, miR-375 targets mitogen-activated protein kinase 8, and as a result, inhibits expression of pro-opiomelanocortin and secretion of pituitary hormones (72). Whether miR-375 is also released by active exocytosis from beta cells and the pituitary gland remains to be determined.

miR-375 is one of the circulating miRNAs in plasma and serum, and might be a biomarker for diabetes (76), hepatocellular carcinoma (77), and Alzheimer’s disease (78). However, it is still under debate as a biomarker, since circulating miRNAs are not disease-specific. Because catecholamines released by LDCV fusion spread through the blood, LDCVs in chromaffin cells can be one source of circulating miR-375, but the function of miR-375 remains to be elucidated in both normal and pathological conditions.

Large dense-core vesicles in neurons might also contain miRNAs. miR-29a and miR-125a are released from synaptosomes in response to depolarization (79), and both miR-29a and miR-125a are among the top 10% most-abundant miRNAs in LDCVs (24). let-7b is also released from dorsal root ganglion neurons by depolarization; let-7b stimulates the toll-like receptor-7 (TLR7)/TRPA1 ion channel to mediate pain signaling (80). let-7b is among the 15% most-abundant miRNAs in LDCVs (24). However, whether these miRNAs are released by LDCV or by synaptic vesicle fusion in neurons remains unknown, as does the physiological function of secreted miRNAs.

## Physiological Functions of Extracellular miRNAs

Even though miRNA exocytosis is selective in response to stimulus in neuroendocrine cells (24), the hypothesis that vesicle-free miRNAs act as signaling molecules and mediate cell-to-cell communication is still challenging. The biological roles of vesicle-free miRNAs remain elusive. Two possibilities are proposed: (1) gene silencing in target cells after endocytosis and (2) cellular signaling by receptor activation.

### miRNA Transport to Regulate Gene Expression in Target Cells

The miRNA transport system between different cells for cell-to-cell communication has been intensively reported including exosomes (81–83), but this review focuses on functional transfer of vesicle-free miRNA. The first evidence of miRNA transport between cells came from plants. Plasmodesmata are channels that traverse the cell walls of plant cells; miRNA are transported directly through plasmodesmata, thus inducing systemic gene silencing of mRNAs in target cells (84, 85).

In the nematode *Caenorhabditis elegans*, SID-1 (a transmembrane channel for dsRNA) and SID-2 (dsRNA transporter) have important functions in uptake of extracellular vesicle-free miRNA into the cytosol; miRNA can be internalized by SID-2-mediated endocytosis and then transported into the cytosol through the SID-1 channel (86, 87). The human SID-1 ortholog SIDT1 facilitates miRNA transfer between human cells (88). In addition to channels of small RNA, miRNA might be transported into the cytosol through HDL receptor SRBI. The complex of HDL with extracellular vesicle-free miRNAs in human plasma binds to SRBI and miRNA delivery might be mediated by a cell surface HDL receptor SRBI (62); however, SRBI-mediated miRNA transfer is still not significant and remains controversial (89). AGO2 and/or NPM1 bind to extracellular vesicle-free miRNAs in cell culture and the protein–miRNA complex might facilitate miRNA uptake into target cells (61, 79). Additionally, secreted miRNAs might be taken up into neurons by endocytosis (79) or through gap junctions in the direct cell contact (90, 91) to regulate the translation of targeted mRNAs. However, little is known about the mechanisms by which extracellular vesicle-free miRNAs can be transported and regulate gene expression in target cells.

### Cellular Signaling *via* Receptor Activation

An unconventional function of miRNA as an agonist of toll-like receptor (TLR) was discovered in 2012: vesicle-free miRNAs interact with TLR7 and TLR8 and, in this way, activate the downstream signaling pathway (92, 93). Tumor-secreted miR-21 and miR-29a stimulate murine TLR7 and human TLR8 in immune cells, and thereby trigger a TLR-mediated inflammatory response (92). Extracellular let-7 activates the TLR7 and induces neurodegeneration through neuronal TLR7 (93). let-7b induces inflammatory pain by activating TLR7 in a sequence-dependent manner as an agonist in dorsal root ganglia (80).

MicroRNAs might evoke cellular signaling by stimulating receptors in the plasma membrane, but the tissue origins of extracellular miRNAs remain unknown. LDCVs in chromaffin cells is one of the origins of extracellular vesicle-free miRNAs, because miR-21, miR-29a, and let-7b are among the 5, 10, and 15% most-abundant miRNAs in LDCVs, respectively (24); this abundance suggests that miRNA may serve as a receptor agonist. However, the binding site of miRNA and receptors is not known at the molecular level. Furthermore, the RNA-binding proteins that can regulate miRNA function to stimulate receptors remain unknown, because free miRNA without protein partners would be highly unstable. Crosslinking protocols can be applied to investigate miRNA-binding proteins that stabilize miRNA after exocytosis.

## Possible Mechanisms of miRNA Loading into LDCVs

Several groups have provided evidence for miRNA sorting into exosomes. Direct contact of miRNAs with MVB membranes might be important for miRNA sorting (94) and universal sequence-specific sorting mechanisms for miRNA loading into EVs have been proposed (95). hnRNPA2B1 protein that recognizes the specific GGAG motif within miRNAs may mediate miRNA sorting into exosomes (96). However, the mechanisms of miRNA sorting into EVs remain largely unclear.

Selective packing of miRNAs to LDCVs is also unknown. Given that miR-375 constitutes ~30% miRNAs stored in LDCVs (24), LDCVs probably uptake miRNAs selectively. Two possible pathways should be considered: (1) because LDCVs in neuroendocrine cells bud off the Golgi complex and undergo maturation (97), miRNAs might be incorporated into LDCVs during biogenesis and (2) RNA transporters in vesicle membranes might uptake miRNA into LDCVs (Figure 1B). However, this hypothesis still requires investigation of how miRNAs are packaged into LDCVs in endocrine and neuroendocrine cells.

## Conclusion

Large dense-core vesicles in chromaffin cells contain miRNAs, which are released in response to stimulation, together with catecholamines and peptides. miRNA exocytosis by the SNARE complex and Syt-1 has been discovered in chromaffin cells and additional secreted vesicle-free miRNAs are expected to be discovered. miRNAs have hormone-like activities; i.e., they are secreted from neuroendocrine cells, spread through the blood stream, and regulate target cells by gene expression and/or cellular signaling. This activity of secreted miRNAs is opening an exciting research area in RNA biology, endocrinology, and neuroscience, but several important questions remain unanswered, including: (1) what are the physiological functions of secreted and vesicle-free miRNAs? It becomes clear that miRNA are secreted by vesicle fusion, but there is little evidence showing the functions of these secreted miRNAs. Further studies should focus on (i) the mechanism of miRNA endocytosis to mediate gene regulation in a target cell and (ii) miRNA receptors that might be activated by secreted miRNAs (Figure 1A). (2) Which cells can release vesicle-free miRNA? In addition to chromaffin cells, other neuroendocrine cells need to be tested whether miRNA is able to be released by vesicle fusion in a SNARE-dependent manner. (3) How can miRNA be stabilized after exocytosis? miRNAs stored in LDCVs are highly stable after exocytosis, but the mechanisms of miRNA stability and which proteins stabilize miRNAs remain to be elucidated. (4) How can miRNA be loaded into LDCVs? ~500 copies of miR-375 are accumulated inside a LDCV, but the loading mechanism of miR-375 remains mysterious. Further studies are required to determine whether there is a miRNA transporter that uploads miRNAs (Figure 1B).

Extracellular vesicle-free miRNAs are considered to contribute to cell-to-cell communication, but the physiological action and the target of extracellular miRNAs remain to be elucidated. In-depth knowledge of extracellular vesicle-free miRNAs will lead to the new research field that miRNAs may behave as hormones to regulate cell-to-cell communication in a paracrine and endocrine manner.

## Author Contributions

YP designed and wrote the manuscript.

## Conflict of Interest Statement

The author declares that the research was conducted in the absence of any commercial or financial relationships that could be construed as a potential conflict of interest.
